# Pro-and anti-inflammatory effects of glucocorticoid Fluticasone on ovarian and immune functions in commercial-aged laying hens

**DOI:** 10.1038/s41598-021-01141-5

**Published:** 2021-11-03

**Authors:** Ali Hatefi, Ahmad Zare Shahneh, Zarbakht Ansari Pirsaraie, Ali Mohammad Alizadeh, Mohammad Pouya Atashnak, Reza Masoudi, Frederic Pio

**Affiliations:** 1 Department of Animal Science, University of Tehran, 77871-31587 Karaj, Iran; 2 Department of Animal Science, Sari Agricultural and Natural Resources University, 48181-66996 Sari, Iran; 3 Cancer Research Center, Tehran University of Medical Sciences, 1419733141 Tehran, Iran; 4 Molecular Biology and Biochemistry Department, Simon Fraser University, Burnaby, BC V5A1S6 Canada; 5 Animal Science Research Institute of Iran (ASRI), Agricultural Research Education and Extension Organization (AREEO), 3146618361 Karaj, Iran

**Keywords:** Animal physiology, Endocrine system and metabolic diseases, Reproductive biology

## Abstract

Ovarian chronic inflammation has been created and extended in the laying hen mainly via increasing laying frequency and microbial infection, especially during the late stage of production period. This study was aimed to evaluate glucocorticoid (GC) Fluticasone as an anti-inflammatory agent on the gene expression of the ovarian pro-and anti-inflammatory mediators (follicular cyclooxygenases COX 1, 2, and cytokines), inflammatory responses of the immune system, ovarian functions (ovulation rate and follicular growths), and hormones in the commercial-aged laying hens. White Leghorn hens aged 92-weeks were used for four weeks to be supplemented by 2 ppm Fluticasone as an optimum dose obtained in a pre-trial base on ovulation rate. As compared to control, Fluticasone resulted in a significant decrease in the mRNA expression of COX-1 and pro-and anti-inflammatory cytokines, and increase in COX-2 mRNA expression and heterophil to lymphocyte ratio (P < 0.001). A significant reduction was observed in the ovulation rate, follicular size (P < 0.001), ovarian hormones, immunoglobulins, body weight, and food consummation (P ≤ 0.05) by administering GC Fluticasone. Although a relative anti-inflammatory improvement was created by Fluticasone in the ovarian condition, the administration of this glucocorticoid resulted in a considerable reduction in ovarian hormones and functions of commercial aged laying hens.

## Introduction

During recent decades, extensive genetic technologies and breeding schedules have improved the production efficacy in the farm animals like commercial laying hens. Nevertheless, this enhancement has remained some reproductive consequences such as chronic inflammation in the laying hens’ ovary compare to the wild birds and native laying hens^1,2^. Besides, the immune system has been shown to influence the inflammatory condition in the ovary through the high frequency of ovulation rate and the spread of microbial infection which accompany with leukocytes infiltration and the production of pro-inflammatory cytokines^3,4^. These could be as the justifiable reasons to contribute to the deterioration of production rate and egg quality in the commercial laying hens^5^ especially, during the late stage of production period^1^.

For recent two decades, several studies demonstrated that the ovarian chronic inflammation was controlled in the aged laying hens by administrating some anti-inflammatory strategies like non-steroidal anti-inflammatory drugs (NSAIDs)^6^, herbal-originated compounds^7^, and the sources of Omega-3 fatty acid^8^ that all of them decreased the ovarian chronic inflammation. Therefore, the evaluation and presentation of different anti-inflammatory strategies may influence the improvement of ovarian inflammation in the aged laying hens. Among these, the usage of glucocorticoids (GC) is current to attenuate inflammatory signs in various diseases like respiratory disorders^9^. However, their inflammatory effects on immune cells and function have been proven through the previous studies that showed that administration of GC hormone or Dexamethasone is followed by dramatic increases in heterophil:lymphocyte (H:L) ratio^10,11^ as a certain indicator in the inflammatory condition^12^.

Despite creating the inflammatory signs in the immune system via GC, the influence of pro-or anti-inflammatory role of GC Fluticasone on the chronic inflammatory responses (ovulation rate, follicular growths, steroids hormones, and mRNA expression of pro-inflammatory cytokines) of the ovary may have either positive or negative correlation on the production efficiency of commercial laying hens during the late stage of production period that their investigation is the authors’ aim in this study.

## Results

### mRNA expression of pro-and anti-inflammatory mediators

**Figure 1:**
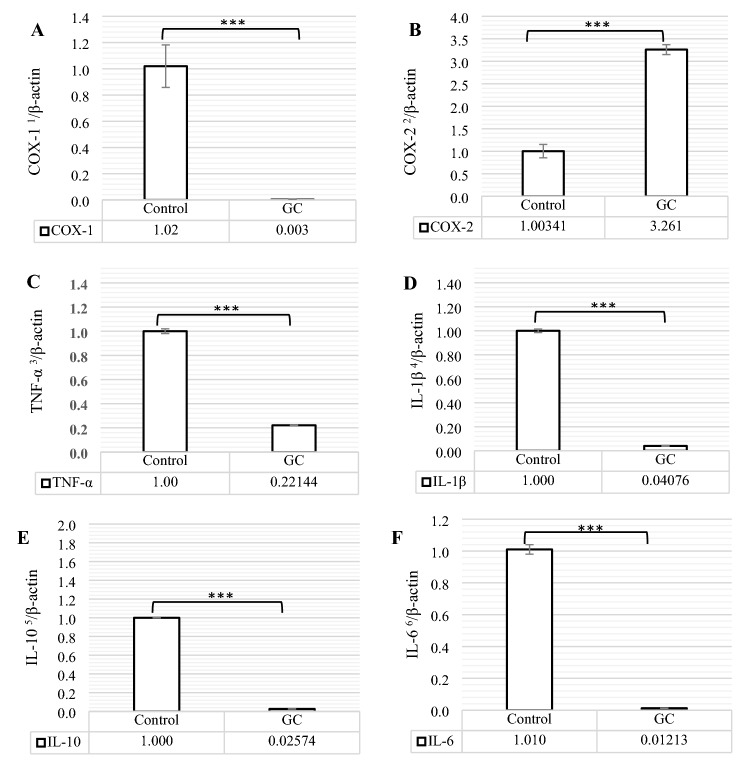
The comparison of COX-1 (**A**), COX-2 (**B**), TNF-α (**C**), IL-1β (**D**), IL-10 (**E**), and IL-6 (**F**) mRNA expressions between control and GC (Glucocorticoid Fluticasone, 2 ppm). Different statistical marks are significant (*P ≤ 0.05, **P ≤ 0.01, and ***P ≤ 0.001) according to the Dunnett's test as a comparison procedure. COX-1, COX-2, TNF-α, IL-1β, IL-10, and IL-6 mRNA data were normalized by β-actin. (1) Cyclooxygenases-1, (2) Cyclooxygenases-2, (3) Tumor necrosis factor-α, (4) Interleukin-1β, (5) Interleukin-10, and (6) Interleukin-6.

The relative abundances of cyclooxygenases 1 and 2 (COX-1 and COX-2) mRNA and the cytokines of interleukin (IL)-1β, IL-6, IL-10, and tumor necrosis factor-α (TNF-α) mRNA in the Pre-ovulatory follicles (F1), normalized via β-actin as a housekeeping gene, were shown in Fig. 1A–F. According to Fig. 1 COX-1, IL-1β, IL-6, IL-10, and TNF-α mRNA abundances were less and COX-2 mRNA expression was higher in GC group when compared to the control group (P < 0.001).

### Analyses of plasma estradiol, progesterone, and androgen

**Figure 2:**
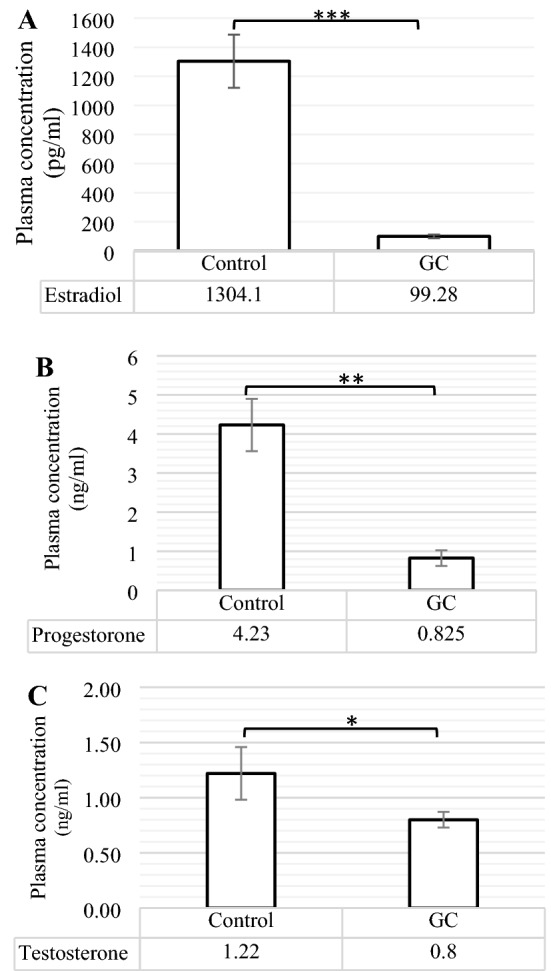
The comparison of plasma Estradiol (**A**), Progesterone (**B**), and Testosterone (**C**) contents between control and GC (Glucocorticoid Fluticasone, 2 ppm). Different statistical marks are significant (*P ≤ 0.05, **P ≤ 0.01, and ***P ≤ 0.001) according to the Dunnett’s test as a comparison procedure.

Figure 2A–C has shown Changes in the plasma concentrations of estradiol, progesterone, and androgen (testosterone) of control and treated laying hens. Compare to control, the hens, supplemented by GC Fluticasone, had less plasma concentration of estradiol (P < 0.001), progesterone (P ≤ 0.01), and testosterone (P ≤ 0.05).

### The function of cellular and humoral immunities

**Figure 3:**
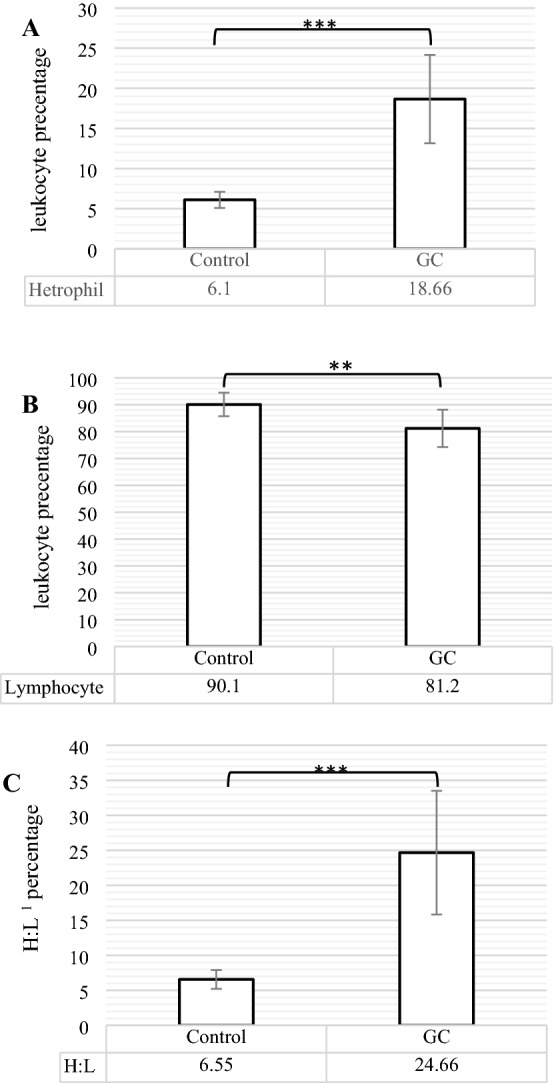
The comparison of heterophil (neutrophil) (**A**), Lymphocyte (**B**), and heterophile: lymphocyte ratio (**C**) between control and GC (Glucocorticoid Fluticasone, 2 ppm). Different statistical marks are significant (*P ≤ 0.05, **P ≤ 0.01, and ***P ≤ 0.001) according to the Dunnett's test as a comparison procedure. (1) Heterophil: lymphocyte ratio.

**Figure 4:**
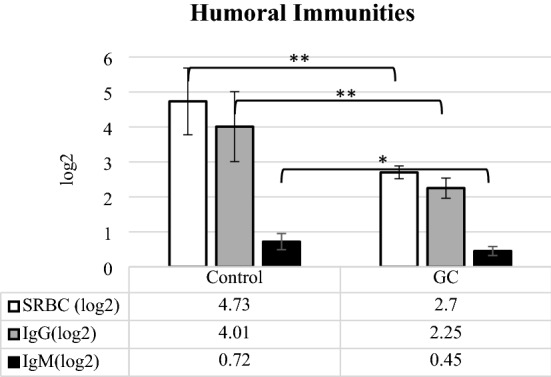
The comparison of whole immunoglobulin (Ig, SRBC), IgG, and IgM contents between control and GC (Glucocorticoid Fluticasone, 2 ppm). Different statistical marks are significant (*P ≤ 0.05, **P ≤ 0.01, and ***P ≤ 0.001) according to the Dunnett's test as a comparison procedure.

Figure 3A–C has presented the changes in neutrophil (heterophil) and lymphocyte percentages, and heterophil to lymphocyte ratio (H:L); and the serum concentrations of immunoglobulins (Ig) G, M, and whole immunoglobulin content or SRBC (Sheep Red Blood Cell, SRBC) have been indicated in Fig. 4. According to Fig. 3, GC group significantly had less lymphocyte (P < 0.01) and higher neutrophil (P < 0.001) percentages when compared to control group. The change of these percentages, observed in GC group, resulted in a significant increase in H:L (P < 0.001) compare to control group. In addition, the birds, administrated by GC Fluticasone, significantly had less serum Immunoglobulin (Ig) G (P < 0.01), IgM (P < 0.05), and consequently whole Ig (P < 0.01) concentrations (Fig. 4) as compared to control group.

### Ovarian and body functions

**Figure 5:**
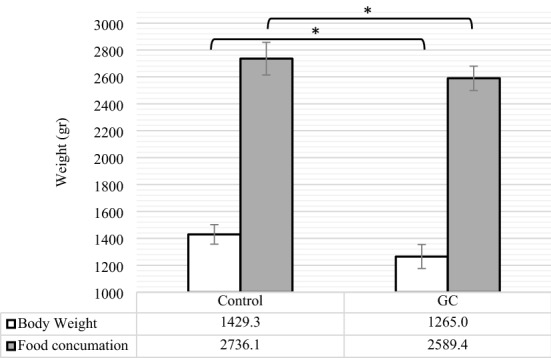
The comparison of body weight and food consummation between control and GC (Fluticasone, 2 ppm). Different statistical marks are significant (*P ≤ 0.05, **P ≤ 0.01, and ***P ≤ 0.001) according to the Dunnett’s test as a comparison procedure.

The changes in hens’ average body weight (BW) and food consummation, as the indicators of body function, have been demonstrated in Fig. 5 and their ovulation rate (laying frequency) and follicle sizes F1 to F5 have been presented in Table 1. According to Fig. 5, average BW and Food consummation were significantly reduced (P < 0.05) in GC group. Results also showed in Table 1 that the ovulation rate was significantly decreased in GC group (P < 0.001) compare to control group. Moreover, the follicular size in the aged hens, supplemented by Fluticasone, were influenced much more than control, because of having smaller diameter (P < 0.001) in follicle F1 than control and lack (not observed, N.O.) of follicles F2 to F5 in these treated hens.

**Table 1:** The comparison of hen’s ovulation rate and follicular sizes F1 to F5 in control and GC groups (mean ± SD).

	Control	GC^b^	SEM^c^	p-value
**Ovulation rate**^**a**^** (%)**	61.55 ± 8.93^d^	33.89 ± 10.27	2.92	***
**Follicular size**				
Follicle F1 (mm)	27.72 ± 5.38	6.25 ± 5.39	2.04	***
Follicle F2 (mm)	27.23 ± 3.16	N.O.^e^	2.07	–
Follicle F3 (mm)	20.85 ± 4.39	N.O	1.91	–
Follicle F4 (mm)	15.54 ± 4.32	N.O	1.79	–
Follicle F5 (mm)	11.44 ± 2.12	N.O	1.36	–

Different statistical marks are significant (*P ≤ 0.05, **P ≤ 0.01, and ***P ≤ 0.001) according to the Dunnett’s test as a comparison procedure.

^a^Laying frequency.

^b^Glucocorticoid (Fluticasone, 2 ppm).

^c^Standard error of the mean.

^d^Standard deviation (SD).

^e^Not observed.

## Discussion

For several decades, GCs have been used in the inflammatory-originated diseases like respiratory and allergic disorders^13^ and systemic autoimmune diseases^14^, for their anti-inflammatory action. In addition, as a glucocorticoid with high receptor affinity and long lung retention, Fluticasone has been known to remain the anti-inflammatory effects on the respiratory disorders like chronic obstructive pulmonary disease (COPD)^15^. GCs remain this property mainly via creating a complex (GC-GR) with their receptors (GR). GC-GR is capable of affecting its anti-inflammatory role thought binding to DNA together with other transcription factors. Moreover, GC-GR has been proven to physically interact with transcription factors without interacting with DNA itself. This is the responsible mechanism for inhibition of the pro-inflammatory transcription factors NFκB (nuclear factor kappa-light-chain-enhancer of activated B cells), activator protein 1 (AP-1), and CREB (cAMP response element-binding protein) which are influenced by the anti-inflammatory and immunosuppressive GCs effects^16^. However, studies suggest that GCs can also exert pro-inflammatory effects in particular organs like the immune system^17^, so that, GC administration could result in the elevation of neutrophil (heterophil in the avian species)-lymphocyte ratio (H:L), the event that was observed in this study and proven by some researches^18^, as the sign of inflammatory condition^19^. According to both of pro-and anti-inflammatory properties of GCs described above, the evaluation of ovarian inflammatory responses such as ovarian hormones, follicular development, ovulation, and mRNA expression of inflammatory mediators could be helpful to confirm the reasons for the negative effect GCs on the production efficiency in the commercial aged laying hens.

As the rate-limiting enzymes, cyclooxygenase (COX) -1 and 2 perform the main role in the various physiological functions and be involved in the different reproductive performances of ovary like ovulation^20^. Even though COX-1 is expressed in a wide range of cells and tissues and stays in the stable expression under the most physiologic situations, COX-2 is an inducible enzyme and generally only produced following various inflammatory situations. Cytokines, on the other hand, which are defined as the products of immune cells, are also expressed by a wide range of non-immune cells, like the ovarian cells; and their function in the ovary has been described as the promoting processes of follicular development, activation of leukocytes required for ovulation, and tissue remodeling during ovulation^21^. Among these, IL-10 as an anti-inflammatory cytokine and IL-6, IL-1β and, TNF-α^22^ as the pro-inflammatory cytokines^23^ play their role in inflammatory reactions. Here, we reported that the administration of GC Fluticasone down-regulated COX-1 and pro-and anti-inflammatory cytokines and up-regulated COX-2 mRNA expression (Fig. 1A–F). Our COX-1 result was in agreement with some reports that demonstrated that glucocorticoids down-regulate COX-1 gene expression^24^; nonetheless, in contrast with our COX-2 result, several documents indicated that COX-2 expression is inhibited by glucocorticoids^25,26^. However, only a few studies reported that glucocorticoid therapy enhances COX-2 expression. In this regard, Sun et al. indicated that GC induced COX-2 gene expression via inducing the interaction of glucocorticoid receptor with C/EBP-β (CCAAT/enhancer-binding protein-β) in cardiomyocytes; in fact, activation of glucocorticoids and their receptors are necessary for COX-2 gene expression due to the binding of both glucocorticoid receptor and C/EBP-β to COX-2 promoter^27^. Sun’s results, explained above, were confirmed by next studies like Adzic et al. that described COX-2 expression is more related to C/EBPβ, so that mutual activation of C/EBP and GR on the COX-2 promoter is required for the increased COX2 expression upon GCs treatment^28^. Our results was agreed with other researches that had documented GC markedly suppressed mRNA expression of key pro-inflammatory cytokines including TNF-a, IL-1β, and IL-6^29,30^, and was unlike others that had mentioned GCs may regulate inflammatory action by increasing IL-10 mRNA expression as well as higher serum IL-10 concentration^31^. Regarding to the GC effects on the cytokines, Cain and Cidlowski described that glucocorticoid-mediated attenuation of cytokine could be a result of inhibiting the expression of many pro-inflammatory cytokines, regulation of cytokine production at the post-transcriptional level, and the attenuation of cytokine receptor signaling^32^ that these three reasons could derive from inhibition of inflammatory transcription factors like NFκB, AP-1, and MAPK (Mitogen-Activated Protein Kinase) pathways, as main factors which are inhibited by glucocorticoids^33–36^. According to the obtained results of decrease and increase in cytokines and COX-2 mRNA expressions, respectively, although pro-inflammatory cytokines have been defined to promote COX-2 production^37^, it seems that the glucocorticoid-mediated attenuation of cytokine could not completely inhibit the elevation of COX-2 mRNA expression promoted by GR—C/EBP-β signaling.

As three ovarian hormones, estradiol, progesterone, and androgens (testosterone) play the functional roles to regulate growth, differentiation, and function of a wide range of target tissues in the females’ genital tract^38^. However, these hormones have been indicated to remain the different inflammatory effects. Some evidence reported that estrogen demonstrates both pro-and anti-inflammatory roles depending on the concentration. In the chronic inflammatory diseases, estradiol inhibits the main pro-inflammatory cytokines like IL-6, IL-1β, and TNF-α at high levels; whereas, the secreted levels of pro-inflammatory cytokines are enhanced during the lower concentrations of estradiol^39^. Progesterone, on the other hand, acts as a protective agent to prevent from inflammation during pregnancy by inhibiting TNF-α and IL-6, and by the recovery of antioxidant enzyme activity in some tissues^40^. Testosterone therapy alleviates the inflammatory process and attenuates the intensity of disease by the mechanisms which inhibit inflammatory cytokines expression and function like IL-6, IL-1β, and TNF-α^41^. About the results expressed in Fig. 2A–C, the birds supplemented by GC Fluticasone, significantly had less plasma estradiol, progesterone, and testosterone, as compared to control group. In keeping with our results, the previous researchers showed that GC remains a negative correlation with ovarian hormones^42,43^. Additional studies demonstrated that reduced concentration of serum estradiol and progesterone in GC group could be derived from the factors like inhibition of hypothalamus–Pituitary–gonads^44^, inhibition of estradiol activity by increasing the expression sulfotransferase^45^, decreasing luteinizing hormone (LH) receptor number^46^, and reduction in ovarian activities as a result of the decrease in BW and food consummation, the events observed in this study. Therefore, according to the anti-inflammatory function of ovarian hormones described above, the administration of GC Fluticasone reduced the anti-inflammatory efficiency of these hormones by decreasing their plasma concentration.

As an inflammatory indicator, H:L has been reported as a certain parameter of the systemic inflammatory response for predicting the situation of various diseases with inflammatory origin^47^. Generally, the factors that enhance inflammatory signs, were followed by higher H:L, and factors inhibiting inflammation, were accompanied by lower H:L^19^. This enhancement of H:L is associated with increasing heterophils and decreasing lymphocytes numbers. Our results demonstrated in Fig. 3A–C that the administration of GC Fluticasone significantly caused to increase heterophil and decrease lymphocyte percentages that were followed by higher H:L as compared to control group. In agreement with our results, some of the documents reported that on the one hand, GC causes not only to elevate the accumulation and survival of neutrophils^48^ but also to up-regulate of anti-apoptotic Bcl-2 family members, activate Nuclear Factor Kappa-light-chain-enhancer of activated B cells (NF-κB), inhibit components of the extrinsic mechanisms of apoptosis, and promote signaling molecules such as MAPK phosphatase-1 (MKP-1) and Serum glucocorticoid activated kinase-1 (SGK-1)^49^ that promote inflammatory aspects in neutrophils. On the other hand, GCs indicated the different behavior on lymphocyte numbers rather than neutrophils because GC results in the skew of T cells, activation of NF-κB via stimulating Toll-like receptors^50^, and activation of death-inducing genes that consequently induce apoptosis in lymphocyte^51^. Observed increase and decrease in heterophils and lymphocytes, respectively, resulted in a rise of H:L in hens administrated by GC Fluticasone that was similar to some studies showing that treatments which used GC, had higher H:L values that are mainly derived from higher neutrophil counts^18^.

Immunoglobulins, As the proteins engaged in anti‐inflammatory reactions, help to send other immune cells to the inflammatory sites, contribute in the anti‐inflammatory processes, and prevent inflammatory reactions^52^. Regarding to main autoantibodies, IgG and IgM were found to have a wide clinical usage as the anti-inflammatory agents in the many autoimmune and inflammatory diseases^53,54^. According to the results shown in Fig. 4, the hens, administrated by GC Fluticasone, had less whole Ig, IgG, and IgM than control group that were in agreement with evidence that proved corticosteroids appear to have a negative correlation on levels of some serum immunoglobulins^55^. Regarding this effect, GC has been reported to decrease B cells activity through promoting intracellular pathways of apoptosis and death-inducing genes^51^, modulating peripheral B cell maturity via inhibiting activation-induced cytidine deaminase (AICDA) expression^56^, dephosphorylation of ERK‐1/2 via increasing dual‐specificity protein phosphatase1 (DUSP1) expression^57^, and down-regulating Bruton Tyrosine Kinase (BTK) for B-cell activation^58^.

Ovulation, considered as an inflammatory-originated phenomenon, has been proven via two hypotheses incessant ovulation (Fathalla’s incessant ovulation hypothesis) and inflammation^2^. According to incessant ovulation, Fathalla has theorized the continuous engagement of the epithelial ovarian surface in the process of ovulation for continuous processes of rupture and repairing the wound of the epithelial surface of the ovary. During the time, these processes boost ovarian chronic inflammation. The inflammation hypothesis, on the other hand, has mentioned that the ovulatory-related events have been demonstrated to resemble an inflammatory reaction that follows with the processes like leukocytes infiltration and production of inflammatory mediators like cytokines, prostaglandins, and the promotion of intracellular mechanisms, closely accompany with inflammatory reaction^59^. Regarding the results, shown in Table 1, the laying hens, supplemented by GC Fluticasone, significantly indicated less ovulation rate and smaller follicle size F1 as compared to control group that. Moreover, Fig. 5 demonstrated that food consummation and BW were reduced in the GC group in comparison with control group. The mentioned results were in line with the documents that indicated feed intake, BW gain, final BW, egg-laying rate, and egg production were all significantly decreased by corticosteroid treatment^11,60^. Besides the effect of inflammatory mediators, the factors such as nutritional-metabolic factors and relevant hormones of the hypothalamus-pituitary-ovary axis perform the essential roles in the functions of follicular growth and ovulation. Regarding the effect of nutritional-metabolic factors, some studies demonstrated nutrients (carbohydrates, fatty acids, and amino acids), energy balance, and metabolic hormones such as growth hormone, insulin, and Insulin-like Growth Factor I (IGF-I) considerably affect the ovarian actions like the follicular development and ovulation^61^. In this regard, some studies proved that using GCs cause to induce Insulin resistance^62^, disturbance of IGF-I^63^, and down-regulation of growth hormone^64^. Therefore, reduced ovulation rate and follicular growth, observed in the GC group, could be derived from (1) decrease in food consummation that resulted in negative energy balance and consequently loss of live BW observed in the GC group, (2) observed reduction in estradiol and progesterone, and (3) observed down-regulation of mRNA expression of inflammatory mediators.

According to the obtained results in this study about the effect of GC Fluticasone administration on down-regulation of cytokines gene expression, authors believe that these down-regulations of cytokines and then the creation of anti-inflammatory condition in the ovary are as a result of two direct and indirect roles of GC on ovary; so that on one hand, via having a negative role to inhibit transcript factors and signaling pathways^33–36^, mentioned above, GC Fluticasone resulted in a direct down-regulation of pro-inflammatory cytokines. On the other hand, Fluticasone, indirectly caused to down-regulation of ovarian cytokines via losing BW as a result of the decrease in the food consummation, observed in this study, and created a negative energy balance. This energy shortage makes a significant dysfunction in the ovarian activities like follicular growth, ovulation, and hormone production^61^, the events observed in this study on the GC treated birds. Because the intensity of follicular growth and ovulation positively relate with the inflammatory condition^59^, decrease in the follicular growth and ovulation may result in the down-regulation in cytokines production in the ovarian follicles.

The results of this study indicated that the administration of 2 ppm per body weight (as an optimum level as the result of a pre-trial) glucocorticoid (GC) Fluticasone caused to down-regulate mRNA expressions of the pro-and anti-inflammatory cytokines and up-regulate cyclooxygenases (COX)-2. GC Fluticasone could create an inflammatory trait via decreasing ovarian hormones and increasing heterophil: lymphocyte ratio (H:L) in the immune system. Ovulation rate and follicular growth were reduced in the hens treated by GC, because of weaker nutritional status and ovarian hormones situation, in addition to the reduction in some pro-inflammatory mediators in the ovary in this group. Taken together, despite creating a relative improvement in ovarian inflammatory condition, the administration of GC Fluticasone brought about a considerable reduction in the ovarian function of commercial laying hens during the late stage of the production period.

## Methods

### Animal care

Thirty-two 92-week-old commercial strains of White Leghorn laying hens *(Gallus domesticus*) were collected and housed at the poultry research station, department of animal sciences, University of Tehran at Karaj. Laying hen husbandry was supervised and approved by the institutional animal care of this department. The laying hens were exposed to a photoperiod program of 16 h light: 8 h dark (light on at 06:00 and off at 22:00), food and water provided ad libitum. Laying frequency (ovulation rate, as one of the ovarian functions), live body weight (BW), and food consummation were monitored and recorded during this experiment. The ingredients and value of the test diet were shown in Table 2.

**Table 2:** The Ingredients (%) and nutrient composition of the diet.

Diets	Value (%)
Corn	61.00
Soybean meal	23.45
Sodium bicarbonate	0.05
D-calcium phosphate	1.53
fatty acid	2.81
Salt	0.07
Calcium carbonate	10.47
Vitamins + minerals	0.50
DL-methionine	0.13
**Calculated analysis**	
Crude protein	15.39
Calcium	4.62
Available phosphorus	0.40
Metabolizable energy	2780^a^

^a^(kcal/kg).

All laying hens were randomly divided and orally supplemented into two groups (*n* = 16) included: control and GC Fluticasone (Jaber Ebne Hayyan Pharma. Co., Tehran, Iran) for four weeks. Optimum supplemented level of Fluticasone (2 ppm body weight, BW), mentioned above, had previously been gained by a pre-trial according to ovulation rate on commercial laying hens with same week-old and supplementing time (92-week-old and four weeks, respectively).

### Blood collection

For evaluating cellular and humoral immunities, and ovarian hormones responses, blood samples (5 ml/hen) were randomly collected from the brachial vein of 10 laying hens per group at the end of four weeks, centrifuged (at 3000 × rpm for 15 min), and their gained serum and plasma were stored at – 20 °C for determination of humoral immune and ovarian hormones, respectively.

### Immune responses

For calculating heterophil to lymphocyte ratio (H:L, as an inflammatory indicator of cellular immunity), Blood samples were smeared on a glass slide. After drying, the blood smears were stained with May–Grünwald–Giemsa stain^65^. The H:L was calculated and obtained by dividing the number of heterophils by the number of lymphocytes. About the evaluation of humoral immunity, on the 14th and 20th day of the Fluticasone administration, all birds were injected by 0.1 mL of 0.25% suspension (in phosphate buffer saline) of sheep red blood cells (SRBC) which was provided from a healthy male sheep. All Anti-SRBC antibody titers of birds’ serum were obtained by micro hemagglutination technique from the samples which were taken from blood collection at the end of the four weeks of Fluticasone administration. Anti-SRBC titers were measured and shown as log2 of the last dilution’s reciprocal after the whole agglutination^66^.

### Ovarian hormones measurement

The concentration of ovarian hormones (estradiol, progesterone, and testosterone) was determined in this study by ELISA (Enzyme-Linked Immune Sorbent Assays) kits (Monobind® Inc, USA), regarding the mentioned manufacturer’s recommendations. The sensitivity of detection, intra-, and inter-assay coefficients of variation (%) for progesterone were 0.105 ng/ml, 1.5% and below 13%, for estradiol were 6.5 pg/ml, 6.3%, and 8.5%, and for testosterone were 0.038 ng/ml, 4.9%, and 4.6%, respectively.

### Tissue sampling

After four weeks, 10 birds were euthanized by CO2 asphyxiation and necropsied per experimental group, then their ovaries were removed and ovarian yellow follicles were arranged base on their diameter (from F1 as pre-ovulatory follicles to F5 as 5th small yellow follicle) which was measured from follicle stigma. After measuring follicular size, Follicles F1 (12–35 mm) were removed from ovaries, washed by saline, kept at microtube, and stored at − 80 °C for RNA isolation^20^.

### RNA isolation and cDNA synthesis

According to the manufacturers’ instructions, total cellular RNA was isolated from frozen tissues via Trizol reagent (RNX-plus, Cinagen Co., Tehran, Iran). The denaturing agarose gel electrophoresis and spectrometric methods were used for the quality and quantity of total RNA, respectively. Before reverse transcription reaction, samples were treated by DNase I (YT 9054, Yekta Tajhiz Azma co., Tehran, Iran) For RNA purification. Then, cDNA was synthesized via the cDNA reverse transcription kit (YT4500, Yekta Tajhiz Azma co., Tehran, Iran). The gained cDNA was stored at − 80 °C for analyzing gene expression using real-time PCR^20^.

### Real-time PCR

Target gene mRNA levels were evaluated by a real-time rotary analyzer (Rotor-Gene 3000, Corbet Research, USA) and SYBR green qPCR master mix (YT 2550, Yekta Tajhiz Azma co., Tehran, Iran).Hen (*Gallus domesticus*) specific primers were gathered in Table 3. β-actin was applied as a housekeeping gene to normalize target gene expression. Amplification conditions: 95 °C for 300 s followed by 50 cycles of 95 °C for 10 s and 60 °C for 30 s with melt curve which was measured at 65–95 °C every 0.5 °C gradient for 5 s. Control reactions lacking template were performed for each target gene. Reactions were 10 μL in total volume and 200 nM for each primer. The relative levels of mRNA expression were analyzed by the 2^−ΔΔC^_T_ method^67^.

**Table 3:** Chicken primers used for real-time PCR.

Gene	Accession no.	Primers sequences (5′ → 3′)	Orientation
COX-1^a^	XM_425326	TCAGGTGGTTCTGGGACATCA	Forward
		TGTAGCCGTACTGGGAGTTGAA	Reverse
COX-2^b^	XM_422297	CTGCTCCCTCCCATGTCAGA	Forward
		CACGTGAAGAATTCCGGTGTT	Reverse
IL-1β^c^	AB559570	CTTCCTCCAGCCAGAAAGT	Forward
		CAGCTTGTAGCCCTTGAT	Reverse
IL-6^d^	AB559572	CAACCTCAACCTGCCCAA	Forward
		GGAGAGCTTCCTCAGGCATT	Reverse
IL-10^e^	AB559574	CACAACTTCTTCACCTGCGAG	Forward
		CATGGCTTTGTAGATCCCGTTC	Reverse
TNF-α^f^	AY765397	TGTGTATGTGCAGCAACCCGTAGT	Forward
		GGCATTGCAATTTGGACAGAAGT	Reverse
β-Actin^g^	L08165	CATCACCATTGGCAATGAGAGG	Forward
		GCAAGCAGGAGTACGATGAATC	Reverse

^a^Cyclooxygenases-1.

^b^Cyclooxygenases-2.

^c^Interleukin-1β.

^d^Interleukin-6.

^e^Interleukin-10.

^f^Tumor necrosis factor-α.

^g^COX-1, COX-2, IL-1β, IL-6, IL-10, and TNF-α mRNA data that are normalized by β-actin.

### Ethics approval and consent to participate

All experiments were performed following relevant guidelines and regulations which had been confirmed by the Ethics Committee of the University of Tehran, and Also, all in vivo experiments were performed in compliance with the ARRIVE guidelines.

### Statistical analysis

Data were analyzed and compared by Dunnett's test using SPSS software (IBM SPSS Statistics, version 26.0, 2019), According to General Linear Model (GLM). The statistical significance of each parameter was investigated as significant at P ≤ 0.05.

## Data Availability

The datasets, generated and/or analyzed during the current study, are available and can be obtained from the corresponding author upon a justifiable request.

## Abbreviations

AICDA: Activation-induced cytidine deaminase
AP-1: Activator protein 1
BTK: Bruton tyrosine kinase
BW: Body weight
COPD: Chronic obstructive pulmonary disease
COX: Cyclooxygenase
CREB: CAMP response element-binding protein
DUSP1: Dual‐specificity protein phosphatase1
ELISA: Enzyme-linked immune sorbent assays
F1: Pre-ovulatory follicles
GC: Glucocorticoid
GC-GR: Glucocorticoid its receptor complex
GLM: General linear model
GR: Glucocorticoid receptor
H:L: Hetrophil to lymphocyte ratio
IGF-I: Insulin-like growth factor I
Ig: Immunoglobulin
IL: Interleukin
LH: Luteinizing hormone
MAPK: Mitogen-activated protein kinase
MKP-1: MAPK phosphatase-1
NF-ƙB: Nuclear factor kappa-light-chain-enhancer of activated B cells
N.O.: Not observed
NSAIDs: Non-steroidal anti-inflammatory drug
ppm: Parts per million
SRBC: Sheep red blood cell
SGK-1: Serum glucocorticoid activated kinase-1
TNF-α: Tumor necrosis factor
VEGF: Vascular endothelial growth factor
